# Covid-fatigued? A longitudinal study of Norwegian older adults’ psychosocial well-being before and during early and later stages of the COVID-19 pandemic

**DOI:** 10.1007/s10433-021-00648-0

**Published:** 2021-08-25

**Authors:** Thomas Hansen, Thomas Sevenius Nilsen, Marit Knapstad, Vegard Skirbekk, Jens Skogen, Øystein Vedaa, Ragnhild Bang Nes

**Affiliations:** 1 Department of Mental Health and Suicide, Norwegian Institute of Public Health, Oslo, Norway; 2 Oslo Metropolitan University, Oslo, Norway; 3 Promenta Research Center, University of Oslo, Oslo, Norway; 4 Department of Health Studies, Norwegian Institute of Public Health, Oslo, Norway; 5 Department of Health Promotion, Norwegian Institute of Public Health, Bergen, Norway; 6 Center for Fertility and Health, Norwegian Institute of Public Health, Oslo, Norway; 7 Department of Psychology, University of Oslo, Oslo, Norway; 8 Alcohol and Drug Research, Stavanger University Hospital, Stavanger, Norway; 9 Department of Public Health, University of Stavanger, Stavanger, Norway; 10 Department of Mental Health, Norwegian University of Science and Technology, Trondheim, Norway; 11 Department of Research and Development, St Olavs University Hospital, Trondheim, Norway

**Keywords:** COVID-19, Older adults, Psychosocial well-being, Loneliness, Norway, Longitudinal analysis

## Abstract

As the pandemic continues, many older adults are facing prolonged isolation and stress while having less access to traditional ways of coping. There is widespread concern that the situation is increasingly taking its toll on older adults’ psychological and social well-being. We use linear mixed models to examine psychosocial impacts and predictors thereof among older Norwegians in early and later stages of the pandemic. Longitudinal data were collected online in the Norwegian Counties Public Health Survey right before the pandemic and in June and November–December 2020 in two counties (baseline *n* = 4,104; age 65–92). Outcomes include loneliness (single item, UCLA3), psychological ill-being (worried, anxious, depressed), and psychological well-being (satisfied, engaged, happy). From before to three months into the pandemic men’s psychosocial well-being remained stable, whereas women’s slightly declined. Five months later we observe broad and substantial declines in psychosocial well-being. These impacts disproportionately affect women (all outcomes) and single and older individuals (loneliness only) and are not moderated by educational level, urbanicity, or whether self or partner are reported “at risk” due to health problems. Pre-pandemic low social support and high psychological distress predict relatively improved psychosocial well-being. Older Norwegians seemed to manage the pandemic’s early stage without clear psychosocial impacts. However, we observe notably compromised well-being during the second wave of COVID-19 in late 2020. Lessons learned about the nature and distribution of the psychosocial impacts of prolonged health-threats and social distancing provide valuable knowledge for intervention design during this and future pandemics.

## Introduction

How are older adults affected by the COVID-19 pandemic, and which subgroups are the most vulnerable? Are there signs of emotional fatigue or increasing loneliness as the pandemic drags on and returns for a second wave in Norway during the fall of 2020? On March 12, 2020, one day after the World Health Organization (WHO) declared COVID-19 a pandemic, the Norwegian government issued several restrictions, including closure of schools, nonessential businesses, and many public facilities (Hansen et al. 2021; The Norwegian Government 2021). While the formal “lockdown” measures were relaxed or removed during the late spring and summer of 2020, the social distancing recommendations remained (e.g., maintain social distance and avoid social gatherings and public transportation). Older adults aged 65 + were singled out as being particularly vulnerable to COVID-19 due to their weaker immune systems and higher likelihood of having a chronic health condition and were urged to take strict distancing measures. Public messaging and media reports soon spread important but potentially anxiety-provoking information to older adults, at the same time issuing warnings of alarming pandemic-related decreases in psychological and social (psychosocial) well-being among older adults. Similar concerns were also raised by mental health scholars (e.g., Killgore et al. 2020) and the WHO (2020).

The pandemic-related health threats and infection control measures have profoundly disrupted daily routines for many older adults and restricted usually pleasurable activities such as visits with friends and family (Bu et al. 2020a). There are several elements of the pandemic that can be a particular source of worry and psychological distress. These include fears or stigmatization due to unclear, somewhat arbitrary age limits regarding who is at particular risk, and the largely uncontrollable health threat with no end date (Kivi et al. 2021). In addition, important mental health impacts could arise from the infection control measures. Many older adults have had limited social contact or access to caregivers and other potentially needed supports (Krendl and Perry 2021). COVID-19 has also compromised opportunities to engage in meaningful and socially valued roles (e.g., grandparenting) that enhance a sense of meaning and purpose in life. Many older adults do not use digital technologies to socially connect, which adds to their vulnerability in the era of COVID-19 (WHO 2020). Prolonged social isolation may thus lower psychological well-being and increase feelings of loneliness—the unpleasant feeling of being isolated from others (Cacioppo et al. 2006).

Meanwhile, many older adults may cope well with the situation and even enjoy aspects of it. First, as shown in past mass tragedies (Calo-Blanco et al. 2017; Saltzman et al. 2020), emerging studies related to the current pandemic suggest that many people experience an enhanced sense of support (Luchetti et al. 2020; Statistics Norway 2020a). Being collectively under threat and going through a shared challenge can promote a sense of solidarity and togetherness (Saltzman et al. 2020). Moreover, older adults may be uniquely able to cope with COVID-19 given their life experiences and coping mechanisms (Fuller and Huseth-Zosel 2020). Gerontological research has long shown that older adults tend to maintain well-being by effectively using secondary coping strategies such as downward adjustment of needs, aspirations, and comparison standards (Brandstätter 2015; Klausen 2020). These strategies promote well-being by fostering smaller aspiration/achievement gaps and are used more often by older than younger adults. Relatedly, aging is associated with gains in competencies to regulate emotional experience. With age there seems to be an increased favoring of positive over negative stimuli even at the level of attention and memory (Carstensen and Mikels 2005). These coping strategies, coupled with experience of previous challenges, predict that older adults may be skilled at identifying positive meaning during the pandemic and feel that “this too shall pass” (Lind et al. 2021).

Older adults constitute a very heterogeneous group, however, and are thus likely to have different reactions to COVID-19. Based on what is known about risks and protective factors during times of crisis (Brooks et al. 2020), pronounced negative effects may be expected among individuals with fewer socioeconomic (e.g., education and income), social (e.g., friendships and support network), and psychological (e.g., emotional stability and sense of control) resources. During COVID-19, specific concern has been expressed for certain subgroups of older adults (Hwang et al. 2020; WHO 2020; Wu 2020). First, for single individuals, who may be particularly isolated and lacking in support during lockdown. Second, for those with an ailing partner, who may face myriad challenges during COVID-19 including secondary worries for the partner’s health, restricted access to visit in care facilities, and denial of relief and home care services. Third, for those who are very old or have pre-existing physical health problems. This group is both more often isolated and lonely prior to the enhanced social distancing (Hansen and Slagsvold 2016) and advised to practice extra-strict social distancing during the pandemic. Fourth, there is concern for those with mental health problems, who may become especially anxious, agitated, or withdrawn during lockdown (Pierce et al. 2020). Finally, given their higher risk profile (e.g., widowhood, health problems, and caregiving), particular concern has been expressed for older women and the oldest old (Hansen and Slagsvold 2016).

Policymakers, services providers, and other stakeholders need reliable information about mental health changes associated with the pandemic across subgroups of older adults. This knowledge is important to develop targeted interventions and to provide support to those most vulnerable, especially as the pandemic continues and new waves of infection could occur (Niedzwiedz et al. 2020). Research has shown that prior large-scale epidemics and disasters have been followed by clear negative changes in psychological well-being for older adults (Brooks et al. 2020; Parker et al. 2016). Preventing similar impacts during COVID-19 is critical given the severity of its scale and associated restrictions. Adding to this importance is the broad and severe consequences of loneliness and psychological problems. These stressors gain prevalence in later life due to widowhood, living alone, or mobility limitations (Hansen and Slagsvold 2016), and are associated with heightened risk of mental and physical illness, cognitive decline, suicidal behavior, and mortality (Holt-Lunstad et al. 2015). Loneliness and mental health were thus recognized as major health concerns among older adults even before the pandemic (WHO 2020).

An emerging literature has begun to document associations between the COVID-19 pandemic and psychological outcomes among older adults, and the findings are mixed (the below review includes only studies of people aged 60 +). Some studies compare cross-sectional data collected before (in 2018/2019) and during the pandemic. Whereas US and Austrian data show increasing loneliness (Luchetti et al. 2020; Stolz et al. 2021; Malani et al. 2020), especially among women (Malani et al. 2020), German data suggest stable levels of loneliness and mental health problems (Röhr et al. 2020). These studies provide limited evidence on causality and on whether outcomes were elicited by, or existed prior to, the pandemic. A handful of studies have used panel data collected right before and during the pandemic, and again the findings are mixed. While a Swedish study (Kivi et al. 2021) shows stable levels of loneliness, US (Krendl and Perry 2021), Swiss (Macdonald and Hülür 2021), and Dutch (van Tilburg et al. 2020) studies find increasing loneliness during the pandemic. Similarly, mental health problems appear stable in a Dutch (van Tilburg et al. 2020) and UK study (Pierce et al. 2020), but increasing in a US study (Krendl and Perry 2021).

This backdrop highlights the need for more longitudinal studies with pre-pandemic data to address changes in psychosocial well-being in response to COVID-19. There is also very limited evidence from non-early stages of the pandemic; at the time of writing the most recent evidence is from the summer of 2020. Hence, it remains unclear whether psychosocial impacts persist, intensify over time, or decrease as people adapt to their new circumstances. Importantly, we lack knowledge about how the population reacted to the second wave of the pandemic during the fall of 2020, when Norway and most other Western countries witnessed a dramatic increase in infection rates and issued stronger infection control measures (Nørgaard et al. 2021). Furthermore, much of the reviewed literature also has other notable limitations that challenge our understanding and suggest avenues for future research, including a use of small or convenience samples, a lack of focus on gender and life stage (young-old vs. old-age) differences, a lack of attention to other risk and protective factors, and the use of only one or two outcomes (Pierce et al. 2020).

The current study examines gender-stratified longitudinal change in loneliness, psychological ill-being, and psychological well-being in a large probability-based sample of older adults surveyed before the pandemic (fall 2019/winter 2020) and then re-assessed twice after formal restrictions had been in place for about three and eight months. To understand heterogeneity in the impacts of the COVID-19 pandemic, we test nine individual-level moderators, including socio-demographic characteristics (age, educational level, employment status, urbanicity), health (subjective health, mental health problems, self or others in the household being at risk of severe COVID-19 illness due to health problems), and social factors (partner status and social support).

## Methods

### Data

The Norwegian Counties Public Health Survey (NCPHS) examines health and quality of life in the Norwegian general population. It is originally a cross-sectional study and invitations are distributed by email and SMS with links to an online survey. Email addresses and cell phone numbers are provided by the registers of the Norwegian Digital Agency. Baseline data (t1) in our study are NCPHS data of community-dwelling individuals aged 18 + collected in Agder (23 Sept-18 Oct 2019, *N* = 28,047, RR = 46%) and Nordland county (27 Jan–16 Feb 2020, *N* = 24,222, RR = 47%). A random sample of 20,103 from these counties was invited to participate in two COVID-19 follow-ups, during 4–18 June (t2; *N* = 11,333, RR = 57%) and 18 November–4 December 2020 (t3; *N* = 10,502, RR = 52%). Agder and Nordland were targeted for the COVID-19 study because they participated in the NCHPS closer in time (< 6 months) to the 12 March 2020 shutdown than other counties. Analyses are based on individuals aged 65–92 at t1 (t1: *N* = 4,104; t2: *N* = 2,865; t3: *N* = 2,831).

### Variables

The NCHPS includes a range of indicators of psychological and social well-being. Psychological well-being refers to subjective well-being, or to how people experience and evaluate their lives (Diener 2012). Social well-being can be defined as an appraisal of one’s social interaction and social relationships, and loneliness is one of its core indicators (Aartsen and Hansen 2020). The NCHPS includes a list of items measuring emotions: “Think about the past 7 days, to what degree did you feel ___?” on a scale from 0 (not at all) to 10 (very). The response format and selection of items conform to conventions and OECD-guidelines in the subjective well-being literature (OECD 2013; Nes et al. 2018). Based on these items, we have constructed two indexes. *Psychological ill-being* is measured by the mean of three items: *worried*, *anxious*, and *down or sad* (*α* = 0.74). *Psychological well-being* is measured by the mean of the items *engaged* and *happy*, and a single item measuring *life satisfaction* (“Overall, how satisfied are you with your life these days?”, measured from 0 to 10) (*α* = 0.74). *Loneliness* is measured with a single item that asks about the degree to which one has felt “lonely” (0–10). We also use the popular and validated three-item version of the UCLA loneliness scale (Hughes et al. 2004), which assesses how often participants report feeling (1) that they lack companionship, (2) left out, and (3) isolated from others. Whereas the original has a 4-point Likert scale, the NCHPS uses a 5-point scale from never (1) to very often (5). The combined scale ranges from 5 to 15 (high loneliness) (*α* = 0.80). We also dichotomized loneliness into “not lonely” (score < 8) and “lonely” (score ≥ 8). UCLA3 was only included in t1 and t3, whereas all other items were included in all three waves.

Demographic variables include *gender*, *age*, *education* (non-tertiary =  < college/university, tertiary = college/university), *partner status* (married/cohabiting = 1, otherwise 0), *employment status* (full/part time, self-employed, or sickness leave = 1, otherwise 0). *Urbanicity* is measured from 1 to 6 (1–4 in our sample as the two most urban levels are not represented in our counties) based on Statistics Norway’s centrality index (Høydahl 2020). *Self-rated health* is recoded into poor (1–2), fair (3), and good (4–5). *Perceived household vulnerability of COVID-19 due to pre-existing health conditions* is measured by whether the respondent perceives themselves or others in the household at risk of severe health consequences if they become infected due to underlying health problems (no/yes). *Psychological distress* is measured using the 5-item Hopkins Symptom Checklist (HSCL-5) (*α* = 0.88) (Strand et al. 2003). The quality of *social support* is measured with the 3-item (e.g., “How many people are you so close to that you can count on them if you have great personal problems”) Oslo Support Scale (OSS-3) (*α* = 0.60) (Meltzer 2003). Scores are categorized into poor (score 3–8), moderate (9–11), and strong (12–14) (Bøen et al. 2012). All independent variables are measured at t1, except perceived health threat (t2).

### Analytical strategy

Mean levels and standard deviations (*SD*) for each outcome are calculated separately for each time point. Change in the outcomes is descriptively assessed by comparing mean values from different time points using t-tests (paired) and Cohen’s *d* (pooled), with effect size 0.2 treated as small, 0.5 as medium, and 0.8 as large (Cohen 1988). To shed additional light on the substantive importance of the observed changes (i.e., how many are “suffering”?), we also show rates of “low” well-being across the three time points. “Low” refers to scores in the undesirable end of the scales, i.e. scores ≥ 6 for negatively worded items (e.g., lonely) and ≤ 4 for positively worded items (e.g., happy).

We use linear mixed models (LMM) with maximum likelihood (ML) estimations and random intercept at the individual level to explore and compare changes in outcomes across time points. Interaction terms of time and group indicate whether changes in outcomes differ across groups. In a first model, all predictors are entered simultaneously, then subsequently each interaction term is added in separate models to avoid multicollinearity. The LMM/ML is a flexible approach for longitudinal analyses that uses all available data under the assumption of missing at random (Enders 2010). Individuals in our sample are nested in municipalities (*n* = 71). However, as the intra-class correlation (ICC) shows that municipality explains less than 1% of the total variance in outcomes, we exclude a random intercept for municipality. We stratify the results in supplementary sensitivity analyses by municipality (Agder vs. Nordland). These analyses are conducted to check regional patterns and potential seasonal effects, as the pre-pandemic data were collected in Sept–Oct in Agder and in Jan–Feb in Nordland. All analyses are stratified by gender and performed using SPSS v. 26.

## Results

The characteristics of the sample at baseline assessment are described in Table 1. A majority (58.2%) are male, the mean age is about 72 years, and around 43% have tertiary level education. The proportions employed (24 vs. 17%) and partnered (83 vs. 68%) are higher among men than women. A substantial proportion report low-moderate level of social support (58–59%), poor health status (29%), psychological distress (14–19%) or that themselves or others in the household have a pre-existing health condition that makes them vulnerable to severe illness from COVID-19 (31%). On a scale from 0 to 10, mean loneliness (1.7–1.8), psychological ill-being (2.6–2.7), and psychological well-being (7.3–7.4) are strongly skewed towards positive (desirable) levels.

**Table 1:** Descriptive statistics. Means (SD) or proportions (%) at baseline (t1)

	Men (*n* = 2,237)	Women (*n* = 1,867)
Age	71.7 (5.2)	70.9 (4.9)
Education (1 = tertiary)	43.6	41.4
Employed (0/1)	23.6	16.8
Urbanicity (1–4)	2.0 (1.1)	2.0 (1.0)
Partner (0/1)	82.5	68.0
Low social support	9.4	8.8
Moderate social support	50.3	49.0
Subj. health (1 = poor)	28.5	28.6
Health threat, self/others (0/1)	30.5	30.7
Psy. distress (1 = high)	14.2	18.7
Loneliness (0–10)	1.65 (2.3)	1.82 (2.26)
Psychological ill-being (0–10)	2.6 (2.1)	2.7 (2.1)
Psychological well-being (0–10)	7.3 (1.6)	7.4 (1.5)

Table 2 shows and compares unconditional means/rates of psychosocial outcomes for the three time points. Change over time is quite consistent across the three indexes and their constituent elements. First, from before to three months into the pandemic (June 2020) men’s psychosocial well-being was quite stable or slightly improving (e.g. “depressed”: –0.25, *p* < 0.01). By contrast, women report slightly higher loneliness (0.16) and psychological ill-being (0.24) and lower psychological well-being (−0.25) three months into the pandemic (*p*’s < 0.01).

**Table 2:** Loneliness and psychological well-being (all outcomes 0–10) before and in the early (June 2020) and later (November–December 2020) stages of COVID-19

	2019/2020 (t1)		June 2020 (t2)		November–December 2020 (t3)		Diff^1^ (Cohen’s *d*)		% with “low” score^2^		
	M	SD	M	SD	M	SD	t1 → t2	t2 → t3	t1	t2	t3
*Men*											
Loneliness	1.51	2.19	1.45	2.18	2.03	2.54	−0.06 (.01)	0.58 (.24)**	7.4	7.5	12.9
UCLA3 Loneliness	1.79	0.69			1.92	0.72		0.13 (.12)**	8.7		12.1
Psychological ill-being	2.21	1.78	2.23	1.69	2.69	1.76	0.02 (.06)	0.46 (.34)**	7.8	4.2	8.9
Worried	2.50	2.44	2.53	2.27	3.27	2.39	0.03 (.05)	0.74 (.29)**	15.4	12.3	18.9
Anxious	1.90	2.31	1.81	2.13	2.31	2.30	−0.09 (.07)*	0.50 (.21)**	10.2	7.8	11.2
Depressed	1.93	2.26	1.68	2.06	2.30	2.29	−0.25 (.17)**	0.62 (.24)**	9.2	7.4	11.5
Psychological well-being	7.50	1.46	7.52	1.40	7.21	1.52	0.02 (.01)	−0.31 (.29)**	3.1	1.9	3.9
Satisfied with life	8.24	1.61	8.26	1.57	7.82	1.72	0.02 (.02)	−0.44 (.30)**	3.3	2.6	3.9
Happy	7.62	1.79	7.61	1.66	7.28	1.76	−0.01 (.01)	−0.33 (.18)**	5.4	4.4	6.1
Engaged	6.66	2.11	6.73	2.04	6.55	2.01	0.07 (.07)	−0.18 (.10)**	12.4	12.0	13.6
*Women*											
Loneliness	1.71	2.18	1.87	2.48	2.72	2.66	0.16 (.05)**	0.85 (.32)**	7.7	10.8	18.0
UCLA3 Loneliness	2.04	0.73			2.26	0.76		0.22 (.20)**	15.2		22.3
Psychological ill-being	2.65	1.81	2.89	1.77	3.51	1.80	0.24 (.31)**	0.62 (.40)**	8.7	8.1	15.1
Worried	3.14	2.47	3.34	2.40	4.18	2.39	0.20 (.16)**	0.84 (.33)**	19.7	19.4	30.8
Anxious	2.36	2.41	2.38	2.34	3.21	2.46	0.02 (.03)	0.83 (.32)**	11.5	11.3	18.5
Depressed	2.36	2.29	2.33	2.369	2.98	2.40	−0.03 (.04)	0.65 (.22)**	11.1	12.1	16.6
Psychological well-being	7.52	1.47	7.27	1.53	6.82	1.57	−0.25 (.30)**	−0.45 (.34)**	2.5	4.3	6.5
Satisfied with life	8.16	1.70	7.97	1.81	7.25	1.86	−0.19 (.17)**	−0.72 (.28)**	4.0	4.5	8.2
Happy	7.69	1.70	7.43	1.79	7.01	1.87	−0.26 (.20)**	−0.42 (.30)**	4.5	5.5	9.9
Engaged	6.71	2.01	6.38	2.11	6.20	1.98	−0.33 (.20)**	−0.18 (.15)**	11.8	15.0	15.8

^1^T-tests. All t1 vs. t3 means *p* < .01

^2^Scores 0–4 for psychological well-being items and ≥ 8 for UCLA3, otherwise scores 6–10. *N* = 1270–2237 (men) and 906–1867 (women)

*p < .05, **p < .01

From June to December 2020, we observe broad and notable declines in psychosocial well-being for both genders but particularly for women (all below *p*’s < 0.01). During this period men and women report higher loneliness (0.58 and 0.78, respectively) and psychological distress (0.45 and 0.62) and reduced psychological well-being (−0.32 and −0.50). From before the pandemic to December 2020, the prevalence of “loneliness” (score ≥ 6) almost doubled for men (from 7 to 13%) and more than doubled for women (from 8 to 18%). These patterns were less pronounced for the UCLA3 scale (from 9 to 12% for men and 15 to 22% for women). Of the psychological ill-being indicators, the largest increase from 2019 to December 2020 is observed for “worried”, where we observe significantly increasing means (from 2.5 to 3.3 among men and from 3.1 to 4.2 for women) and rates (from 15 to 19% for men and from 20 to 31% for women). Of the well-being indicators, the most notable component change was a strong decline in women’s life satisfaction by almost a full point (0–10 scale) from before the pandemic (8.1) and in June 2020 (8.0) to November–December 2020 (7.2).

Table 3 shows the results of mixed linear modeling of change in psychosocial outcomes. The analyses examine potential predictors of psychosocial outcomes and change in these outcomes. Random intercept-only models show that differences *between* persons account for 49% of the total variance in loneliness (intraclass correlation (ICC) = 2.87/[2.87 + 3.00]) and ill-being (ICC = 2.16/[2.16 + 2.26]), and 60% of the variance in well-being (1.51/[1.51 + 1.00]). The upper half of Table 3 shows that being married/cohabiting (loneliness and well-being), high social support, good health, and low psychological distress are associated with favorable psychosocial well-being. Turning to the altogether nine tested interactions, we find more increased loneliness associated with being older, unpartnered, having high (vs. low) social support, and high psychological distress. Furthermore, adverse psychological changes (increased ill-being or decreased well-being) are associated with higher levels of social support, higher self-reported (subjective) health, and high levels of psychological distress. Changes in outcomes are unrelated to educational level, urbanicity, and “at risk” status due to health problems. In gender-collapsed models (ancillary analyses, not shown) we find significant interactions between gender and time variables, indicating the women (ceteris paribus) report more adverse changes in the three outcomes.

**Table 3:** Linear mixed models. Unstandardized estimates (*B*) and standard errors (SE)

	Men			Women		
	Loneliness	Ill-being	Well-being	Loneliness	Ill-being	Well-being
Time2 (June 2020)^1^	−0.02 (0.05)	−0.13 (0.05)**	−0.09 (0.02) **	0.17 (0.07)*	0.05 (0.05)*	−0.24 (0.04) **
Time3 (November–December 2020)	0.52 (0.06)**	0.53 (0.05)**	−0.40 (0.03)**	0.97 (0.08)**	0.83 (0.07)**	−0.62 (0.04)**
Age	0.02 (0.01) *	0.01 (0.01)	−0.01 (0.00) *	0.01 (0.01)	0.00 (0.01)	-0.01 (0.00)
Education (1 = tertiary)	−0.11 (0.08)	−0.04 (0.05)	0.05 (0.04)	−0.08 (0.09)	−0.06 (0.05)	0.03 (0.04)
Employed (0/1)	0.09 (0.10)	0.11 (0.08)	0.01 (0.06)	0.09 (0.13)	0.01 (0.10)	0.15 (0.09)
Urbanicity (1–4)	0.05 (0.04)	0.03 (0.02)	0.01 (0.02)	0.08 (0.04)	−0.03 (0.03)	0.03 (0.03)
Partner (0/1)	−1.52 (0.10)**	−0.09 (0.08)	0.42 (0.07)**	−1.18 (0.10)**	−0.01 (0.06)	0.34 (0.07) *
Low social support	1.24 (0.15)**	0.29 (0.12) *	−1.01 (0.10) **	1.82 (0.17) **	0.87 (0.11)**	−1.24 (0.07)**
Moderate social support	0.54 (0.08)**	0.25 (0.10)**	−0.48 (0.05)**	0.76 (0.10)**	0.46 (0.10)**	−0.55 (0.04)**
Subj. health (1 = poor)	0.60 (0.06)**	0.44 (0.07)**	−0.68 (0.06)**	0.58 (0.06)**	0.21 (0.06)**	−0.53 (0.07)**
Health threat, self/others (0/1)	0.05 (0.08)	0.19 (0.06)**	−0.07 (0.05)	0.19 (0.09)*	0.15 (0.07)**	−0.13 (0.07)*
Psy. distress (1 = high)	1.85 (0.09)**	2.08 (0.07)**	−1.24 (0.06)**	1.80 (0.11)**	2.31 (0.09)**	−1.39 (0.07)**
*Interactions*						
Age*time2	0.02 (0.01)*			0.04 (0.02)*		
Age*time3	0.02 (0.01)*			0.04 (0.02)**		
Partner*time2	−0.30 (0.11)*			−0.41 (0.10)**		
Partner*time3	−0.26 (0.12)**			−0.33 (0.12)**		
Low support*time2	−0.37 (0.18)*	−0.85 (0.21) **	0.52 (0.12)**		−0.70 (0.22)**	0.27 (0.11)*
Low support*time3	−0.57 (0.23)*	−0.77 (0.18)**	0.67 (0.13)**	−0.64 (0.20)*	−1.28 (0.24)**	0.53 (0.16)**
Moderate support*time2			0.23 (0.07)**			
Moderate support*time3			0.15 (0.07)*		-0.40 (0.14)**	0.18 (0.09)*
Poor health*time2		−0.26 (0.11)*	0.30 (0.07)**		−0.30 (0.13)**	0.30 (0.09)**
Poor health*time3		−0.24 (0.11)*	0.16 (0.08)*		−0.27 (0.14)*	0.26 (0.10)**
High distress*time2	−0.43 (0.12) **	−1.10 (0.11)**	0.57 (0.07)**	−0.40 (0.16) *	−0.85 (0.14)**	0.37 (0.09)**
High distress*time3	−0.29 (0.13)*	−1.08 (0.13)**	0.36 (0.08)**	−0.46 (0.19) *	−1.18 (0.16)**	0.43 (0.11)**

Main effects analyzed without interaction terms in the model. Each interaction term analyzed in separate models (with all main effects). For brevity, non-significant interaction terms are not presented. ^1^Ref = Time1. *N* = 2237 (men) and 1867 (women)

*p < .05, **p < .01

## Discussion

This study provides a descriptive portrait of longitudinal trends in three common indicators of psychosocial well-being (loneliness, psychological ill-being, and psychological well-being) before the pandemic and after three and eight months into the pandemic among older adults in two Norwegian counties. During the early-middle stage of the pandemic, in June 2020, we find no indication of a general upsurge in psychosocial problems, especially among men. In fact, overall men in this sample report slightly increased well-being at this stage. Women, meanwhile, report slight declines in psychosocial outcomes. Prior studies of older adults yield mixed results. Based on a unique and rich dataset, we echo those observing no or only minor psychological changes in the early stages of COVID-19. At least three interpretations can be offered. First, that findings reflect and highlight resilience during the lockdown. This interpretation resonates with extensive research demonstrating the human capacity to adapt to adverse life situations. Partly, this adaptation stems from the stabilizing influence of dispositional factors (Fujita and Diener 2005): changing life circumstances may affect well-being for a while, but over time it tends to fall back to its stable—or baseline—level, determined by genes and personality traits (Mund et al. 2020). Older adults may also show unique resilience during the pandemic based on age differences in stress reactivity and coping resources, and their wealth of life experience to draw upon (Lind et al. 2021; Losada-Baltar et al. 2021). Second, the lack of strong emotional impacts in June 2020 likely also reflects the low infection rates and the relief of infection control measures in this period compared to the preceding months in Norway (NIPH 2021). Finally, the findings likely speak to heterogeneity among older adults. The mean-level stability may disguise significant variations in people’s individual experience of the lockdown: some may have felt anxious and confined, while others may have felt safe and have appreciated needing to slow down.

Eight months into the pandemic, in November–December 2020, we observe broad and substantial negative changes in psychosocial well-being, and again women seem disproportionately affected. For example, while the rate of loneliness was about 7–8% for both genders before the pandemic, it increased to 18% for women and 13% for men in November–December 2020. Similarly, we observe a reduction in life satisfaction by almost one point among women. This drop corresponds to more than half the standard deviation and is similar to the average short-term drop in life satisfaction observed following unemployment or widowhood (Clark et al. 2008). Especially if the pandemic-related stressors become prolonged these drops merit attention from a public health perspective as even small increases in loneliness and mental distress may detrimentally impact on physical and mental health problems (Hawkley and Cacioppo 2010; Holt-Lunstad et al. 2015). Note also that the reductions are likely underestimated, as the oldest age group in large surveys tend to be skewed towards higher functioning older adults, especially in online surveys (Hansen and Slagsvold 2012). In addition, the study does not include institutionalized and frail elderly, whose well-being may be particularly compromised during lockdown. Over time, social distancing and uncontrollable and pervasive stressors such as those related to COVID-19 thus seem to take their toll on psychosocial functioning. Whereas older adults initially report stable well-being, over time, when negative psychosocial experiences accumulate and intensify, individuals may lack the coping resources to maintain high well-being. While, as argued, the observed adverse changes are substantial and important, it should be recognized that the levels of psychosocial well-being (e.g., mean life satisfaction of 7.5) among older Norwegians in late 2020 is still higher than those found among their counterparts in most other Western countries even in normal times (Helliwell et al. 2018).

We examined a range of factors that potentially could moderate the impacts of COVID-19 and signalize risk and protective factors. First of all, women’s psychosocial well-being is significantly more impacted by COVID-19 than men’s. This vulnerability goes beyond women’s higher risk profile regarding widowhood and health problems in older age (Hansen and Slagsvold 2016; Wenham et al. 2020). We can only speculate as to possible explanations, but one may be gender differences in social expectations. Insofar as women generally are more socially active and integrated (Hansen and Slagsvold 2016; Pinquart and Sörensen 2006), social distancing may lead to a larger relative social deficit. Similarly, women of this generation tend to take on greater family care responsibility (ibid.), and the lockdown may have caused greater disruption to their social relationships and valued roles (e.g., as grandparents), which in turn may foster dissatisfaction and loneliness. Furthermore, and contrary to popular beliefs, being single, very old, or in poor physical or mental health do not seem to represent critical risk factors. While singlehood and higher age predict more loneliness during COVID-19, they are unrelated to changes in well-being and ill-being. The link between pandemic-related loneliness and old age, shown also in US panel data (Luchetti et al. 2020), is expected given that older adults in particular have been advised to self-isolate, and many do not communicate digitally. Findings furthermore demonstrate no or minor independent effects on psychosocial outcomes of either educational level, employment status, or self-reported health risk during COVID-19. This pattern may reflect heterogeneity within groups; for example, some people with health problems (e.g., immune deficiency) may strongly self-isolate, whereas others may be largely unaffected or even feel more supported and integrated during the pandemic.

One particularly noteworthy finding is the favorable trajectories in psychosocial well-being among groups with pre-pandemic high levels of psychological distress, social isolation, and loneliness. These patterns run counter to UK population data showing that loneliness worsened among the already lonely, but improved among the not lonely (Bu et al. 2020b). However, because of the strong correlation between initial status and change, and the related floor effects and regression towards the mean, it is expected that the most favorable change would occur among those initially the most distressed (Kelly and Ye 2017). It is also important to recognize that these groups, while reporting relatively favorable changes, still report disproportionately high distress and loneliness both before and during the pandemic. Nonetheless, the beneficial changes observed in the abovementioned disadvantaged groups are noteworthy, counter-intuitive, and at odds with the notion that people with pre-existing high levels of psychological distress would be particularly vulnerable and need extra support during the pandemic (Killgore et al. 2020). Their relative improvement in psychosocial well-being may reflect that actual or perceived increase in social and emotional support during the pandemic (Luchetti et al. 2020; Statistics Norway 2020a) may be particularly potent for those with high loneliness and distress before the pandemic. Other interpretations may be that social distancing represents a comparably larger and more distressing life change for people with strong social relations, and that their (pre-pandemic) lonelier and more distressed counterparts have more experience with some of the stressors associated with the COVID-19 pandemic. Finally, people with normally high access to social support may find it difficult to access this support due to the distancing measures.

This study has several strongpoints, most notably a within-person design and recent data which enable assessment of trajectories also across later stages of the pandemic. A further strength is the scope of variables and the large sample size, providing rich possibilities for moderation analysis. The reliance on online questionnaires contributes to mitigate social desirability bias and improve reliability when probing about sensitive issues (Hansen and Slagsvold 2016). At the same time, however, these methods are likely to miss populations especially vulnerable during the pandemic, such as the oldest old and people living in long-term care facilities.

There are some other caveats and limitations to note. First, issues pertain to the generalizability of our findings. While the response rates of the individual waves can be considered satisfactory, there may be non-random patterns of participation and attrition. While the timing and subject of the follow-up studies may have attracted individuals who were particularly distressed, dropout is normally skewed towards the most distressed (Hansen and Slagsvold 2012). The latter is also suggested by ancillary analysis of our data, as dropouts had higher loneliness (mean 1.89) at t1 than those who participated in all three waves (1.66). Furthermore, the sample is skewed towards the highly educated and younger individuals, a common pattern for online surveys. For example, of the Norwegian population aged 67 + , 27% of men and 21% of women have tertiary education (Statistics Norway 2020b), against 39 and 41% in our sample. These patterns may affect the overall means and proportions to a degree but should have less effect on moderation analyses. Second, findings should be interpreted in light of the relatively non-restrictive lockdown and few COVID-19 cases and related deaths in Norway. Coupled with the relatively generous welfare supports and favorable health and socioeconomic conditions among older adults in Norway, pandemic-related distress could be different, and probably greater, in other countries. Third, as we only have data from two counties, we do not know how generalizable the results are to Norway as a whole. The included counties are rather rural. Urban areas, especially the capital of Oslo, has had higher infection rates and stricter control measures. That said, the issued government restrictions were largely national, especially at the beginning of the pandemic, and this could negate strong regional patterns of pandemic-related psychological impacts. Fourth, there is no way that we can test if data are missing at random (MAR) and not “missing not at random” (MNAR). However, we use a method for handling missing data (ML) which is broadly recommended for handling missing data in longitudinal analysis and is superior to, e.g., listwise deletion (which assumes MCAR) (Enders 2010). Fifth, seasonal changes in outcomes may confound the results as the sample was followed from fall/winter (t1), spring (t2), to fall (t3). However, a number of studies have found no or very weak evidence for seasonal fluctuations in mood or psychological problems (Øverland et al. 2020). Also, our findings are similar in the two counties despite that fact that t1 data were collected in Sept–Oct in Agder and Jan–Feb in Nordland. Finally, there are potential weaknesses related to the use of single-item measures and unvalidated scales. Although our individual single-item measures are commonly used and recommended measurements in the field, the composite indexes should be validated in future research. While findings are remarkably uniform across indexes and constituent elements, thus suggesting that the choice of scale construction make little difference, the constituent elements may not adequately cover the full range of psychosocial experiences.

Despite these limitations, and as was its central aim, this study highlights potentially important public health implications and makes a number of contributions to international scholarship on impacts of COVID-19. First, the emerging pattern from this analysis is, within an older, Norwegian population, one of resilience and adaptation in the summer of 2020 and one of increasing strain—or emotional fatigue—later in the fall, when restrictions had been in place for eight months and the pandemic was on the rise rather than coming to an end. In an international perspective, the magnitude of the adverse psychosocial impacts observed among older adults are particularly noteworthy as they emerge in regions with relatively low COVID-19-related morbidity and mortality rates—in a country with already low such rates. On top of this, older Norwegians may benefit from relatively high levels of trust and social cohesion, favorable financial and health status, and extensive social welfare and health care systems (Hansen and Slagsvold 2016). The observed negative impacts are stronger among women than men, but surprisingly uniform across other social groups. Interestingly, and as described anecdotally by several psychotherapists (Probst et al. 2020), people with pre-existing psychosocial problems as a group report somewhat reduced loneliness and improved well-being during COVID-19. Lessons learned from this study of psychosocial impacts of protracted social isolation and pandemic-related health stressors can inform risk stratification and targeted intervention strategies at both clinical and community level during the ongoing and possible future pandemics or times of crisis.

## Data Availability

Available on request from the authors.
